# Novel Targeted Agents in Hodgkin and Non-Hodgkin Lymphoma Therapy

**DOI:** 10.3390/ph8030607

**Published:** 2015-09-17

**Authors:** Natalie S. Grover, Steven I. Park

**Affiliations:** Division of Hematology/Oncology, Department of Medicine, University of North Carolina at Chapel Hill, Chapel Hill, NC 27599-7305, USA

**Keywords:** Hodgkin lymphoma, non-Hodgkin lymphoma, monoclonal antibody, antibody-drug conjugates, immune-checkpoint inhibitors, small molecule inhibitors

## Abstract

There has been a recent emergence of novel targeted agents for treatment of Hodgkin and non-Hodgkin lymphoma. In particular, antibodies and antibody-drug conjugates directed against surface antigens, agents that block immune checkpoint pathways, and small molecule inhibitors directed against cell signaling pathways have shown significant promise in patients with relapsed and refractory disease and in the frontline setting. With the development of these new therapies, cytotoxic chemotherapy may be avoided entirely in some clinical settings. This review will present the latest information on these novel treatments in Hodgkin and non-Hodgkin lymphoma and will discuss both recently approved agents as well as drugs currently being studied in clinical trials.

## 1. Introduction

Lymphoma is the most common hematologic malignancy and there will be an estimated over 80,000 new cases of both Hodgkin (HL) and non-Hodgkin lymphoma (NHL) in 2015 and over 20,000 deaths. NHL is the 6th most common type of cancer in both males and females accounting for 5% and 4% of new cancer cases, respectively [1]. Survival rates for both HL and NHL have significantly improved over the past several decades [1], but patients with relapsed and refractory disease continue to do poorly. Treatment options for patients with lymphoma were limited to cytotoxic chemotherapy and radiation in the past, but the recent introduction of novel targeted agents has started to greatly impact the field. This review will discuss novel antibodies and antibody-drug conjugates directed against surface antigens, antibodies directed against immune checkpoint proteins, and small molecule inhibitors directed against cell signaling pathways (Table 1).

**Table 1:** Overview of novel agents discussed in this review and their targets.

Drug	Target
Monoclonal Antibodies	
Obinutuzumab	CD20
Ofatumumab	CD20
Epratuzumab	CD22
Lucatumumab	CD40
MEDI-551	CD19
Antibody Drug Conjugates	
Brentuximab vedotin	CD30
Polatuzumab vedotin	CD79b
Inotuzumab ozogamicin	CD22
SAR3419	CD19
SGN-CD19A	CD19
Bispecific T-cell Engager	
Blinatumomab	CD19/CD3
Immune Checkpoint Inhibitors	
Ipilimumab	CTLA-4
Pidilizumab	PD-1
Nivolumab	PD-1
Pembrolizumab	PD-1
MPDL3280A	PDL-1
Small Molecule Inhibitors	
Ibrutinib	BTK
Idelalisib	PI3Kδ
Duvelisib	PI3Kγδ
TGR-1202	PI3Kδ
Copanlisib	PI3Kαδ
INCB040093	PI3Kδ
INCB039110	JAK1
Pacritinib	JAK2
Navitoclax	Bcl-2
Venetoclax	Bcl-2
Alisertib	Aurora kinase A

## 2. Novel Antibodies and Antibody-Drug Conjugates Directed against Surface Antigens

**Figure 1:**
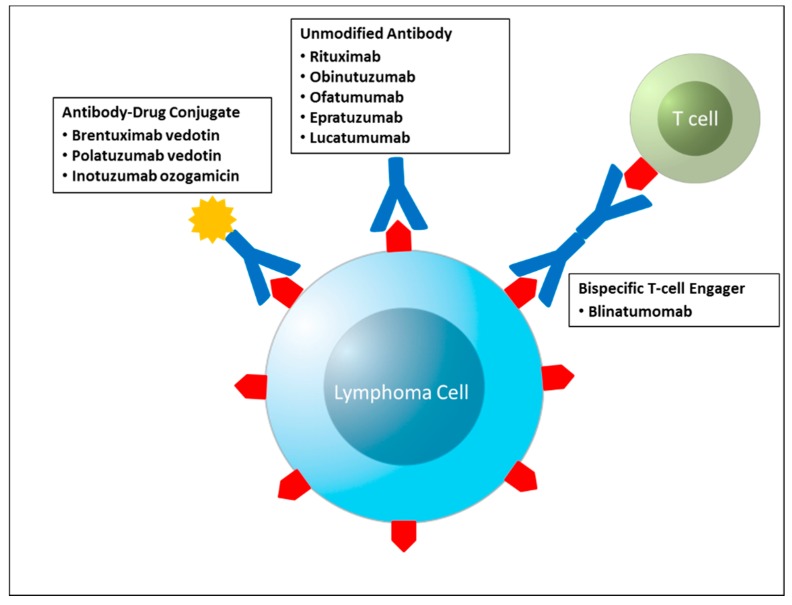
Schematic representation of the antibody based therapies discussed in this review.

Rituximab, a chimeric monoclonal antibody to CD20, became the first monoclonal antibody approved by the FDA in 1997 and initially was only approved for relapsed or refractory indolent lymphoma. It is now used as a single agent, in conjunction with cytotoxic chemotherapy, and for maintenance therapy in B-cell NHL. The addition of rituximab has improved survival in patients with NHL [2,3,4]. Since the remarkable success of rituximab, many antibodies against cell surface antigens have been developed to further improve clinical outcomes in patients with both NHL and HL (Figure 1).

### 2.1. Obinutuzumab (Anti-CD20)

Although rituximab has been very successful in NHL, novel anti-CD20 antibodies have been developed in the hopes of improving the cytotoxic mechanisms of rituximab and potentially overcoming mechanisms of resistance to rituximab in relapsed and refractory disease.

One of the most promising novel anti-CD20 agents is obinutuzumab, a glycoengineered monoclonal antibody. The Fc portion of obinutuzumab is glycoengineered and its fucose content is reduced which increases its affinity to the FcγRIIIa receptor on immune effector cells causing increased antibody-dependent cellular cytotoxicity [5]. Also, obinutuzumab has type 2 antibody binding which has been shown to lead to direct cell death in a process separate from apoptosis [6].

Obinutuzumab was found to be well tolerated in phase 1 studies with the most common adverse events being infusion-related reactions which usually occurred during the first infusion and resolved with treatment [7,8].

The results of the prior phase 1 trials were promising and led to phase 2 trials to further investigate the efficacy of obinutuzimab. The phase 2 GAUGUIN study enrolled both patients with indolent [9] and aggressive [10] relapsed/refractory NHL and randomly assigned them to two different dosing regimens for eight cycles. In the 40 patients in the indolent NHL group, the overall response (OR) rate was 55% (95% CI, 32.2% to 75.6%) in the higher dosing group (1600 mg on days 1 and 8 of cycle 1 and 800 mg on day 1 of subsequent cycles) and 17% (95% CI, 3.6% to 41.4%) in the lower dosing group (400 mg on days 1 and 8 of cycle 1 and then on day 1 of subsequent cycles). In the 40 patients in the aggressive NHL group, the OR rate was 32% (80% CI, 18% to 49%) in the higher dosing group and 24% (80% CI, 12% to 40%) in the lower dosing group. More than half of the patients in both studies (22 out of 40 in the indolent NHL group, 25 out of 40 in the aggressive NHL group) were rituximab refractory. The study did not have enough power to investigate for statistically significant differences between the two treatment arms but it suggested that the higher dosing schedule was more effective, with responses seen even in patients refractory to rituximab, and still well tolerated, with the most common side effect being infusion related reactions, the majority of which were grade 2 or lower.

Given the promising anti-lymphoma activity and favorable safety profile of obinutuzumab monotherapy, the phase 1b GAUDI trial evaluated the safety and efficacy of obinutuzumab in combination with chemotherapy in patients with follicular lymphoma (FL) [11,12]. Obinutuzumab was found to be well tolerated with promising activity in combination with cyclophosphamide, doxorubicin, vincristine, and prednisone (CHOP) and fludarabine and cyclophosphamide in patients with relapsed/refractory FL [11] and in combination with bendamustine and CHOP in the first line setting [12]. The most common adverse events were cytopenias and infection.

The efficacy of obinutuzumab was also investigated in rituximab-refractory patients with CD20+ NHL in the phase III GADOLIN study which compared bendamustine alone with bendamustine and obinutuzumab in 396 patients [13]. Patients on the bendamustine and obinutuzumab arm who did not have progressive disease received maintenance therapy with obinuzutumab for up to two years. The median investigator-assessed and independent radiology facility progression-free survival (PFS) were 14 months and 14.9 months, respectively for the bendamustine arm and 29 months and not reached, respectively for the obinutuzumab and bendamustine arm. The results of this study highlight obinutuzumab as a promising agent for patients with rituximab-refractory disease.

The first comparison of obinutuzumab to rituximab in 175 patients with relapsed or refractory indolent NHL (129 with FL) showed higher response rates in patients randomized to receive obinutuzumab compared to rituximab [14]. Patients treated with obinutuzumab did have a higher rate of infusion related reactions but these were mostly grade 1 or 2 and did not result in a significant difference in discontinuation. Obinutuzumab was also compared head to head with rituximab in patients with untreated chronic lymphocytic leukemia (CLL). In a phase 3 randomized trial, obinutuzumab combined with chlorambucil demonstrated superior PFS and response rates compared to rituximab combined with chlorambucil [15]. The results of this study helped prompt approval of obinutuzumab by the FDA for treatment of CLL in combination with chlorambucil.

Obinutuzumab has been shown to be well tolerated and has promising anti-lymphoma activity both alone and in combination with chemotherapy, even in patients who have been refractory to rituximab, and has been evaluated in the first line, maintenance, and relapsed/refractory setting. There is currently a phase 2 study comparing obinutuzumab and rituximab in the first line setting in untreated indolent NHL (NCT01889797). There is also a phase 3 trial comparing obinutuzumab combined with chemotherapy to rituximab combined with chemotherapy in patients with untreated advanced stage indolent NHL (NCT01332968).

### 2.2. Ofatumumab (Anti-CD20)

Ofatumumab is a humanized monoclonal antibody which targets a novel epitope of CD20 and has been shown to be more effective in complement-dependent cytotoxicity than rituximab in pre-clinical studies [16,17]. Ofatumumab was initially studied in patients with relapsed/refractory CLL and, given the promising results in this population, it was approved by the FDA for treatment of CLL refractory to fludarabine and alemtuzumab [18,19]. A recent randomized phase 3 study investigating the combination of ofatumumab and chlorambucil in patients with CLL unfit for fludarabine-based therapy found improved response rate and PFS with this combination with manageable side effects [20]. These results led to the FDA approval of ofatumumab in combination with chlorambucil for patients with CLL not eligible for fludarabine-based therapy.

Ofatumumab has also been studied in patients with relapsed/refractory FL and was found to be well tolerated but with modest activity as single agent therapy [21,22], leading to the study of this agent in combination therapy. In a phase 2 study investigating the combination of ofatumumab and CHOP in patients with untreated FL, the OR rate and complete response (CR) rate were similar to those previously reported for R-CHOP, but the rate of hematologic toxicity, specifically grade 3–4 neutropenia, was 90%, which is higher than that reported historically for R-CHOP [23]. Ofatumumab has also recently been studied in combination with bendamustine in patients with untreated indolent B-cell NHL and was found to have comparable activity to historical results for bendamustine and rituximab [24].

Ofatumumab has also been studied in diffuse large B-cell lymphoma (DLBCL). A phase 3 randomized trial compared the efficacy of ofatumumab *versus* rituximab in combination with dexamethasone, cytarabine, and cisplatin (DHAP) in 447 patients with relapsed/refractory DLBCL [25]. There was no significant difference in response, progression free or overall survival, or toxicity between the two arms. The activity of ofatumumab has been less promising in NHL than in CLL. Other novel anti-CD20 agents are currently being investigated for patients with relapsed or refractory NHL [26].

### 2.3. Brentuximab Vedotin (Anti-CD30)

CD30 is expressed on several subtypes of lymphoma, most notably anaplastic large cell lymphoma (ALCL) and Reed-Sternberg cells in classical HL. Because its expression in normal cells is limited to activated B and T cells, it is a desirable therapeutic target. However, initial studies with monoclonal antibodies targeting CD30 had limited success [27]. Brentuximab vedotin (BV) is an anti-CD30 monoclonal antibody which is linked to the antimicrotubule agent monomethyl auristatin E (MMAE). The release of MMAE into the cell when the antibody drug conjugate (ADC) binds to CD30 causes disruption of the microtubule network and cell cycle arrest and apoptosis. BV was found to be effective in pre-clinical mouse xenograft models with ALCL and HL [28], which are two conditions with poor prognosis after relapse and for which more targeted therapies are needed [29,30].

A phase 1 dose-escalation study investigating the safety and activity of BV in 45 heavily pretreated patients with CD30 positive hematologic malignancies (42 with HL) showed that the agent had promising activity with objective responses seen in 17 patients (11 CR) with moderate adverse events, the most clinically significant being peripheral neuropathy, seen in 22% of patients [31]. A phase 2 study investigated the safety and efficacy of BV in patients with relapsed or refractory HL after autologous stem cell transplant and showed an OR rate of 75% (95% CI, 64.9% to 82.6%) with 34% of patients achieving CR (95% CI, 25.2% to 44.4%) and median duration of response for patients in CR of 20.5 months [32]. Based on the results of this trial, BV was approved by the FDA for treatment of patients with HL who have either failed autologous stem cell transplant or two other chemotherapy regimens and are not eligible for transplant.

Given the promising results of BV in patients with relapsed and refractory HL, a phase 1 trial investigated BV in combination with chemotherapy in 51 patients with newly diagnosed HL [33]. The results showed that BV combined with ABVD (doxorubicin, bleomycin, vinblastine, dacarbazine) had a high rate of pulmonary toxicity (44%), but the BV and AVD (without bleomycin) combination was generally well tolerated with a 96% CR rate (95% CI, 79.7% to 99.9%) in 25 patients. Currently, a phase 3 trial is in progress comparing BV plus AVD to ABVD as frontline therapy in advanced HL (NCT01712490) which could redefine HL therapy.

A pivotal phase 2 trial explored the activity of BV in 58 patients with relapsed and refractory ALCL [34]. The OR rate was 86% (95% CI, 74.6% to 93.9%) and CR rate was 57% (95% CI, 43.2% to 69.8%) with median response duration lasting greater than one year in this high risk population where 72% of patients had anaplastic lymphoma kinase (ALK) negative disease and 26% of patients had treatment failure after autologous stem cell transplant. The excellent response in this trial led to the accelerated approval of BV by the FDA for the treatment of relapsed or refractory systemic ALCL after failure of at least one prior multi-agent chemotherapy regimen. A recent update to this study showed an impressive 4 year survival rate of 64% (95% CI, 51% to 76%) with 47% of the patients in CR still not showing evidence of progression and 10 out of 17 patients receiving a consolidative stem cell transplant [35].

Given the impressive response seen with BV monotherapy in relapsed disease, a phase 1 study evaluated the safety and activity of BV in combination with chemotherapy as first line therapy in CD30+ peripheral T cell lymphoma and showed that combining BV with CHP (vincristine omitted to reduce neurotoxicity) had promising activity and a tolerable safety profile [36]. Currently, a double blind, randomized phase 3 trial (ENCHELON-2) comparing BV + CHP *vs*. CHOP in newly diagnosed CD30+ T cell lymphoma is in progress (NCT01777152).

BV has shown remarkable effectiveness in both ALCL (as well as other peripheral T cell lymphomas) and HL. Current phase 3 trials are in progress comparing BV in combination with chemotherapy to conventional chemotherapy both in HL and ALCL, the results of which may dramatically change frontline therapy for both of these agents, similarly to rituximab in B-cell NHL. In addition, studies which have shown lack of correlation between efficacy of BV and CD30 expression [37] have opened the avenue of studying the role of BV in other lymphomas. There is currently a phase 1 trial investigating the safety and effectiveness of BV and rituximab as frontline therapy in patients with CD30 positive or EBV positive lymphomas (NCT01805037). BV has also shown activity in DLBCL. The interim results of a phase II study investigating the combination of BV with R-CHOP in first line treatment of patients with high-intermediate/high-risk DLBCL were recently reported and showed promising efficacy with an overall response rate of 97% with 80% PET-negative CR [38]. The CR rate was higher in CD30+ patients than in CD30− patients. The study has been modified to focus on patients with CD30+ DLBCL and is currently in progress (NCT01925612).

### 2.4. Polatuzumab vedotin (Anti-CD79b)

The success of BV encouraged investigation of other ADCs with improved linker technologies for lymphoma treatment. CD79B was felt to be a good target because of its expression on most B cell malignancies. Polatuzumab vedotin is an ADC that contains an anti-CD79B monoclonal antibody which, like BV, is conjugated to the microtubule disrupting agent MMAE. In a recently published phase 1 study, 95 patients with relapsed or refractory NHL and CLL were treated in dose escalation cohorts to determine the maximum tolerated dose and assess safety and tolerability of the drug [39]. The most common grade 3–4 adverse events were neutropenia, anemia, and peripheral sensory neuropathy. Objective responses were noted in 23 of 42 patients with NHL treated at the recommended phase 2 dose (7 CR and 16 PR) with a median PFS of 5.7 months. No objective responses were observed in patients with CLL. The combination of polatuzumab vedotin with rituximab was also evaluated and found to have an acceptable safety profile. Seven of nine patients treated with polatuzumab vedotin combined with rituximab showed an objective response (2 CR and 5 PR) with a median PFS of 12.5 months. This study showed encouraging activity of polatuzumab vedotin in a high risk, heavily pretreated population as well as the possibility of combining this agent with rituximab containing regimens. There is currently a clinical trial studying the replacement of polatuzumab vedotin for vincristine in R-CHOP in the first line setting for DLBCL (NCT01992653).

### 2.5. Epratuzumab (Anti-CD22)

CD22 is also a promising target for antibody therapy since it is expressed by most B-cell NHLs. Epratuzumab is a humanized monoclonal anti-CD22 antibody that has shown promising activity in NHL. A phase 1/2 dose escalation study investigated the safety and efficacy of epratuzumab in 56 patients with relapsed or refractory aggressive CD22+ NHL (35 patients with DLBCL) and showed an OR rate of 15% (95% CI, 5% to 32%) in DLBCL patients with a good safety profile [40]. Studies investigating the safety and efficacy of combining epratuzumab and rituximab showed improved results with an OR rate of 67% in DLBCL (4 of 6 patients, 3 CR) and 63% in indolent NHL (10 of 16 patients, 9 CR) in one study [41] and an OR rate of 47% in aggressive and indolent B-cell NHL (30 of 64 patients, 14 CR) in another study [42]. Given the positive responses with rituximab and the fact that rituximab improves efficacy in CHOP therapy, it was postulated that the addition of epratuzumab to R-CHOP in the front-line setting could increase response rates. A phase 2 trial tested the efficacy of epratuzumab plus R-CHOP in 106 patients with untreated CD22+ DLBCL [43]. The OR rate was 96% (74% CR). This study showed impressive response rates but there were high rates of neutropenia (85% grade 3 or 4 neutropenia). In addition, patients were selected to be positive for CD22, and the efficacy of epratuzumab is unclear in patients with lower or no CD22 expression.

### 2.6. Inotuzumab ozogamicin (Anti-CD22)

CD22 is internalized upon antibody binding which makes it an excellent target for ADC. Inotuzumab ozogamicin (INO) is an ADC which involves the conjugation of a humanized anti-CD22 antibody to calicheamicin, which is a potent cytotoxic antibiotic. A phase 1 study of 79 patients with relapsed and refractory CD22+ B-cell NHL was conducted to determine the maximum tolerated dose, safety, and efficacy of INO [44]. The most common adverse event was thrombocytopenia which was primarily reversible. The objective response rate was 68% (95% CI, 45% to 86%) for patients with FL and 15% (95% CI, 4% to 35%) for patients with DLBCL treated at the maximum tolerated dose. Given the promising results and tolerability seen in this heavily pretreated population, a phase 1/2 study evaluated the safety and efficacy of combining rituximab and INO in 118 patients with relapsed/refractory B-cell CD22+ NHL [45]. The OR rate was 87% in FL, 74% in relapsed DLBCL, and 20% in refractory aggressive NHL. The safety profile was similar to that of single agent INO with thrombocytopenia being the most common toxicity. Given the promising results of this trial, a phase 3 trial was conducted which compared INO-rituximab to bendamustine-rituximab or gemcitabine-rituximab in relapsed and refractory aggressive NHL [46]. This study has been discontinued because the primary objective of improving overall survival (OS) was not achieved.

### 2.7. Lucatumumab (Anti-CD40)

CD40 is a desirable target for lymphoma therapy since it is expressed on the majority of cell surfaces in B cell malignancies and is thought to have a greater range of expression than CD20. Lucatumumab is a humanized anti-CD40 monoclonal antibody. A phase 1/2 trial evaluated the safety and efficacy of lucatumumab in 111 patients with relapsed and refractory HL (37 patients) and NHL (74 patients) [47]. This trial showed that the drug is well tolerated with a promising OR rate of 33% for patients with FL. Further study of this agent potentially in combination with other agents is indicated.

### 2.8. MEDI-551 (Anti-CD19)

CD19 plays an important role in the B-cell receptor pathway and is expressed at an earlier stage than CD20 in both normal and malignant B cells so it is another desirable target for treating B-cell lymphoma. MEDI-551 is a humanized IgG anti-CD19 monoclonal antibody which has been affinity-optimized and afucosylated to enhance antibody dependent cellular cytotoxicity. In a phase 1/2 dose escalation and expansion study, 91 patients with relapsed or refractory CLL, DLBCL, FL, or multiple myeloma (MM) received MEDI-551 [48]. The drug was well tolerated and no maximum tolerated dose was found with the most common Grade 3/4 adverse event being neutropenia. This was a heavily pretreated population with a median of six lines of prior therapy. The OR rate for MEDI-551 was 24% in CLL, 24% in DLBCL, and 31% in FL (9 CR and 12 PR). The median PFS was around nine months. A phase 1 dose escalation study was conducted in Japan with 20 patients (6 with DLBCL, 11 with FL, 2 with CLL, 1 with MM) enrolled and again showed acceptable tolerability of the agent with promising response in refractory B-cell lymphoma patients [49]. There is currently a phase 2 randomized trial of MEDI-551 *vs*. rituximab in combination with salvage chemotherapy in patients with relapsed or refractory DLBCL (NCT01453205).

### 2.9. SAR3419 (Anti-CD19)

CD19 is a desirable target for ADC therapy because it is quickly internalized upon antibody binding [50]. SAR3419 is a novel ADC which combines the humanized anti-CD19 monoclonal antibody with the tubulin inhibitor DM4. In a phase 1 trial of SAR3419, 39 patients with relapsed CD19+ B-cell lymphoma were treated with escalating doses of SAR3419 on an every 3 week schedule [51]. This was a heavily pre-treated patient population with a median of four prior treatment regimens with 11 patients having prior autologous or allogeneic stem cell transplants. The dose limiting toxicities were microcystic epithelial corneal changes resulting in reversible blurry vision (6 patients) and neuropathy (1 patient). Hematologic toxicity was low. In the patients with ocular toxicity, vision returned to baseline within 3 days and the epithelial corneal changes resolved as well. There were 35 patients who completed at least two cycles of therapy and these patients were evaluated for tumor response. Twenty six patients (74%) showed a reduction in tumor size with six patients achieving partial or complete remission. Seven out of 15 patients with rituximab-refractory disease showed a reduction in tumor size. This study demonstrated the safety of SAR3419 and showed promising clinical activity in a difficult to treat patient population, including patients with disease refractory to rituximab. A second phase 1 trial evaluated a weekly administration schedule of SAR3419 to see if more frequent administration at lower doses increased tolerability and effectiveness [52]. This study determined an optimal schedule of four weekly infusions followed by four biweekly infusions which showed an improved safety profile and preserved efficacy. The OR rate was about 30% with about half of rituximab refractory patients showing a response. This data supports the further investigation of SAR3419 by itself and in combination with other therapies in B-cell lymphoma.

### 2.10. SGN-CD19A (Anti-CD19)

SGN-CD19A is another novel ADC composed of anti-CD19 monoclonal antibody conjugated to monomethyl auristatin F (MMAF), a microtubule disrupting agent. Forty four patients with relapsed or refractory B-cell NHL (89% with DLBCL) were enrolled in a phase 1 study investigating the safety and antitumor activity of SGN-CD19A [53]. An interim analysis showed an OR rate of 30% (95% CI, 17% to 46%) with a 16% CR rate. There were no dose limiting toxicities although more than half of patients showed corneal exam findings which seem to respond to ophthalmic steroids and dose modifications. The low rate of hematologic toxicity and neuropathy as well as the level of response in relapsed and refractory patients is promising for potential use in combination with other therapies in an earlier setting. This trial is still in progress.

### 2.11. Blinatumomab (Anti-CD19/CD3 Bispecific)

Another method to target CD19 expressing tumor cells is via blinatumomab, which is a bispecific T cell engager antibody construct which has specificity for both CD19 and CD3 antigens, with the goal of engaging the CD3-expressing cytotoxic T cells to lyse CD19 expressing tumor cells [54]. A phase 1 study investigated the clinical activity and safety of blinatumomab as a continuous IV infusion over 4–8 weeks in 38 patients with relapsed or refractory B-cell NHL and showed 11 major responses with tumor regression observed in patients with FL, mantle cell lymphoma (MCL), and CLL, with most patients showing durable response [55]. The most common side effects were pyrexia, lymphopenia, leucopenia, chills, and increase in C-reactive protein and these mainly occurred during the first week of treatment and subsequently improved or normalized. There were also rare central nervous system (CNS) side effects noted, including encephalopathy, speech disorders, tremors, and confusion, which were reversible with discontinuation of treatment. A phase 2 study evaluated the efficacy of blinatumomab in 21 patients with relapsed/refractory DLBCL with an OR rate of 43% and median duration of response of 11.6 months, which shows promise in this high risk population [56]. The drug has also shown remarkable activity in acute lymphoblastic leukemia (ALL) with a CR rate of 43% (95% CI, 36% to 50%) in a relapsed/refractory population [57], prompting the FDA to grant accelerated approval for blinatumomab for treatment of relapsed/refractory Philadelphia chromosome negative B-cell precursor ALL. Blinatumomab is now primarily being studied and used in patients with ALL, but more investigations need to be performed to further delineate the role of the agent in B-cell NHL, especially given the promising results above in relapsed/refractory aggressive lymphomas.

The success of both monoclonal antibodies and ADCs has greatly changed the treatment terrain in both NHL and HL, improving survival in patients both in the first line and in relapsed/refractory settings.

In addition, the development of bispecific T-cell engagers, such as blinatumomab, introduces an entirely new field of antibody-based immunotherapy which stimulates a patient’s own immune system to attack cancer cells. Chimeric antigen receptor (CAR) T-cell therapy, which uses autologous infusion of genetically engineered T cells that express chimeric antigen receptors targeting surface antigens, such as CD19, is currently being investigated for lymphoma treatment with promising preliminary data. Further discussion of this new treatment modality is outside the scope of this review, but the study of CAR T-cells has gained scientific interest and we expect to see many clinical trials investigating its role in lymphoma therapy over the next few years.

## 3. Novel Antibodies Directed against Immune Checkpoint Proteins

The immune system plays an important role in controlling and eliminating cancer. Tumor cells often produce negative signals which can inhibit the host’s antitumor immune response. Novel agents developed to block immune checkpoint pathways which contribute to tumor tolerance, particularly Cytotoxic T-lymphocyte-associated protein 4 (CTLA-4) and Programmed cell death-1 (PD-1), have been shown to be effective in solid tumor oncology and have now been investigated in hematologic malignancies [26].

These agents also come with a unique pattern of immune-related adverse events including gastrointestinal toxicities (such as diarrhea and colitis), pneumonitis, transaminitis, rashes, and endocrinopathies [26].

CTLA-4, which is a member of the CD28/immunoglobulin superfamily, plays an important role in the immune checkpoint pathway by negatively controlling the function of regulatory T cells leading to a decrease in antitumor immune response [58]. It was the first immune checkpoint receptor to be targeted. PD-1 is another negative regulator of T cell function which inhibits signaling pathways that activate T cells when it interacts with two ligands, PD-L1 and PD-L2 [26,59]. There are many new monoclonal antibodies that inhibit the PD-1 axis, either by inhibiting PD-1 or PD-L1, which have been studied in different cancers including lymphomas. Classical HL has been of particular interest as an ideal target for anti-PD1 therapy [60]. Studies have identified that Reed-Sternberg (RS) cells use the PD-1 pathway to bypass immune detection. Alterations in chromosome 9p24.1 in HL cause overexpression of PD-1 ligands, PD-L1 and PD-L2, on RS cells. The extended 9p24.1 amplification region also includes the JAK2 locus and JAK2 amplification, through the JAK2/STAT pathway, increases PD1 ligand expression [60]. In addition, EBV infection also increases PD-1 ligand expression in EBV-positive HL [59,61]. These findings suggest that HL has a strong dependence on the PD-1 pathway for survival and targeting this pathway could be an effective method of treating the disease.

### 3.1. Ipilimumab (Anti-CTLA-4)

Ipilimumab is a monoclonal antibody that targets CTLA-4 and was initially shown to have significant clinical benefit in solid tumors such as melanoma [62]. Given the success and safety of the agent in solid tumor trials, a phase 1 study of ipilimumab was performed in patients with relapsed and refractory B-cell NHL [58]. Eighteen patients with relapsed and refractory B-cell NHL (14 FL, 3 DLBCL, 1 MCL) were treated with ipilimumab and found to have an 11% response rate (1 patient with DLBCL with CR and one patient with FL with PR). Although the clinical response rate was relatively low, the patients who did show a response showed a durable response, with the response for the FL patient lasting 19 months and the DLBCL patient lasting over 31 months. The durable response seen with single agent therapy stimulated continued interest in investigating immune checkpoint blockade in hematologic malignancies.

There is currently a phase 1 trial investigating the combination of ipilimumab and rituximab in patients with relapsed or refractory B-cell lymphoma (NCT01729806).

### 3.2. Pidilizumab (Anti-PD1)

Pidilizumab is a humanized anti-PD1 monoclonal antibody. In a phase 1 study involving patients with advanced hematologic malignancies, it was shown to be safe and well tolerated with some evidence of clinical activity including a durable CR in a patient with FL [63]. A phase 2 study evaluated the efficacy of pidilizumab in patients with DLBCL after autologous stem cell transplant [64]. The post autologous stem cell transplant population was chosen for study because it is a state characterized by low volume disease during which there is remodeling of the immune system with prevalence of lymphocyte subsets which are targets of PD-1 blockade. The investigators’ hypothesis was that early post-transplant PD-1 inhibition could prevent a decrease in anti-tumor lymphocytes mediated by PD-1 and cause eradication of minimal residual disease. Sixty six patients with relapsed DLBCL were treated with three doses of pidilizumab starting 1-3 months after transplant. The 16 month PFS was 72% (90% CI, 60% to 82%) among all 66 patients. The 16 month PFS was 70% (90% CI, 51% to 82%) in 24 patients who had a positive positron emission tomography (PET) scan prior to transplant at the completion of salvage therapy which compared favorably with a similar historical control population. In patients who had measurable disease after transplant, the response rate with pidilizumab treatment was 51% with a 34% CR rate. Pidilizumab was well tolerated with the most frequent grade 3 or 4 adverse events being neutropenia (19%) and thrombocytopenia (8%). Of note, there was no evidence of significant autoimmune toxicity. There is currently a phase 2 trial in progress investigating the efficacy of pembrolizumab (another PD-1 inhibitor, see below) after autologous stem cell transplant in relapsed/refractory DLBCL and classical HL (NCT02362997).

A phase 2 trial also investigated the combination of pidilizumab and rituximab in relapsed FL [65]. The group hypothesized that combining pidilizumab and rituximab would have a synergistic anti-tumor effect by activation of both the NK cell and T cell-mediated immune response. Nineteen out of 29 (66%) evaluable patients with relapsed rituximab-sensitive FL achieved an objective response with 52% (15 patients) noted to have CR. The median time to response was 88 days and there were many delayed responses with 21% of patients responding more than four months after the first pidilizumab infusion. The median PFS was 18.8 months. The combination of rituximab and pidilizumab was found to be safe without any grade 3 or 4 treatment related or autoimmune adverse events. The one caveat of this study was that it excluded patients with rituximab-resistant disease. This study has established the safety of combining pidilizumab and rituximab and encourages further study of immune checkpoint blockade in FL.

### 3.3. Nivolumab (Anti-PD1)

Nivolumab is a humanized anti-PD1 monoclonal antibody which has shown promising results in the treatment of solid tumors. A phase I study of nivolumab in patients with relapsed or refractory hematologic malignancies including classical HL, B-NHL, T-NHL, and multiple myeloma was recently performed. The results for the 23 patients with HL were recently published [66]. The HL study consisted of a high risk population with a median of 4–5 lines of prior systemic therapy with 78% of patients relapsing post BV and 78% of patients relapsing post autologous stem cell transplant. The OR rate was 87% (95% CI, 66% to 97%) with a 17% CR rate. The remaining three patients on study had stable disease. The median follow up time of this study was short but many of the responses appeared durable with some patients in continued remission for over a year. The drug also showed an acceptable safety profile despite the fact that this was a heavily pre-treated patient population. Drug-related grade 3 events which were reported in five patients (22%) included myelodysplastic syndrome, pancreatitis, pneumonitis, stomatitis, increased lipase level, and decreased lymphocyte count. There were no grade 4 or 5 events. Overall, nivolumab was found to be well tolerated and have impressive activity in patients with relapsed or refractory HL. A phase II registration study of nivolumab in patients with classical HL who have relapsed post autologous stem cell transplant has recently completed enrollment (NCT02181738).

The results for the rest of the lymphoid malignancies in this phase I trial were also recently reported [67]. These patients were also heavily pre-treated with more than two thirds of patients receiving greater than or equal to three prior treatment regimens. Drug-related serious adverse events were uncommon but present with 7% of patients with B-cell NHL experiencing pneumonitis. The OR rate for patients with B-cell NHL was 28% (7% CR) with an OR rate of 36% in patients with DLBCL and 40% in patients with FL. The OR rate was 17% in patients with T-cell NHL (no CR) with an OR rate of 40% in the five patients with peripheral T-cell lymphoma. There is currently a phase 2 trial investigating the efficacy of nivolumab in relapsed or refractory FL (NCT02038946) and relapsed or refractory DLBCL (NCT02038933).

### 3.4. Pembrolizumab (Anti-PD1)

Pembrolizumab is another humanized anti-PD1 monoclonal antibody. The KEYNOTE-013 study investigated the safety and efficacy of pembrolizumab in patients with relapsed or refractory hematologic malignancies. The results of the classical HL cohort of this study, which included patients who had relapsed from or failed to respond to prior BV treatment with two thirds also failing prior autologous stem cell transplant, were recently reported [68]. There were no serious adverse events. The most common drug-related adverse events were grade 1–2 respiratory events (20%) and thyroid disorders (20%). The OR rate was 53% in 15 patients evaluated so far with a 20% CR rate. The results for this high risk population are encouraging. This trial is still in progress with final results including data from the B-cell lymphoma arm of the study still pending. A phase II registration study of pembrolizumab in relapsed or refractory classical HL has recently opened (NCT02453594).

The encouraging safety and efficacy profile of PD-1 inhibitors in relapsed and refractory HL raises interest in using PD-1 inhibitors earlier in HL treatment and in combination with other therapies.

Current studies are investigating the efficacy of pembrolizumab as consolidation therapy post autologous stem cell transplant in DLBCL and HL (NCT02362997), in combination with rituximab in relapsed FL (NCT02446457), and in combination with chemotherapy for advanced lymphoma (NCT02408042).

### 3.5. Other Therapies

Other immune checkpoint inhibitors are currently in investigation. Lymphocyte-activation gene-3 (LAG-3) is another immune checkpoint which is currently being targeted in a phase 1 study in hematologic malignancies, including lymphoma (NCT02061761). In addition, MPDL3280A, an anti-PDL1 antibody, in combination with obinutuzumab is being evaluated in patients with relapsed/refractory FL and DLBCL (NCT02220842).

The promising preliminary results in immune checkpoint inhibition in patients with refractory HL and NHL in conjunction with a good safety profile suggest that these medications will play an important role in future treatment of lymphoma.

## 4. Novel Small Molecule Inhibitors

The B-cell receptor signaling pathway is important for B cell proliferation and survival. An early actor in the B-cell receptor signaling cascade is Bruton tyrosine kinase (BTK), a member of the Tec kinase family. Mutations in BTK in humans cause Bruton’s agammaglobulinemia which is characterized by absence of mature B cells and low immunoglobulin levels [69].

Phosphoinositide 3-kinase (PI3K) is another pathway involved in the B-cell receptor signaling cascade which acts downstream of BTK. PI3K has several tissue specific isoforms (alpha, beta, gamma, and delta). The delta isoform is expressed in hematopoietic cells and plays an important role in B-cell development and function. The PI3Kδ signaling pathways are often overexpressed in B-cell malignancies which has raised interest in their inhibition [70,71].

The Janus kinases (JAKs) are a family of intracellular non-receptor tyrosine kinases which are phosphorylated and activated when a cytokine binds to their associated receptors, which triggers recruitment and phosphorylation of STAT (signal transducers and activators of transcription) proteins. The phosphorylated STAT proteins form a dimer and translocate to the nucleus, where they activate transcription of an array of target genes involved in a variety of processes including cell proliferation and immunity. The abnormal activation of the JAK-STAT pathway has been linked to many malignancies, including lymphomas, which has raised interest in targeting the pathway [72].

The BCL-2 family of proteins are important regulators of apoptosis. Pro-survival BCL2 proteins are often overexpressed in lymphoma which has encouraged the search for BCL2 inhibitors. However, this has been difficult and early agents targeting BCL2 were not as effective as expected, potentially because the BCL2 family of proteins has been found to be redundant. Recently, new agents have been developed which do not directly inhibit BCL2 but instead act as mimics of BH3, which inhibit BCL2 and related proteins [73,74].

The Aurora kinase family controls the mitotic phase of the cell cycle and aurora A kinase (AAK) has been found to play an important role in mitosis [75]. Overexpression of AAK has been identified in various malignancies, including lymphoma [76], and has been shown to cause malignant transformation in mouse models, establishing its oncogenic role [77]. Inhibition of AAK causes mitotic arrest [78].

### 4.1. Ibrutinib (BTK Inhibitor)

Ibrutinib is an oral selective irreversible small-molecule BTK inhibitor which inhibits B cell receptor signaling by occupying the active site of BTK. In a phase 1 dose escalation trial, ibrutinib was found to be well tolerated with an OR rate of 60%, with the highest response seen in patients with MCL and CLL/SLL [69].

Given the promising results of the phase 1 trial, a phase 2 trial examined the efficacy of ibrutinib in relapsed and refractory MCL in 111 heavily pre-treated patients who had received a median of three prior therapies [79]. The OR rate was 68% (CR 21%) with a median response duration of 17.5 months. This study also found a unique toxicity of grade 1 and 2 bleeding in 17% of patients and subdural hematomas in four patients which were associated with fall or head trauma. The patients who had subdural hematomas had all received aspirin or warfarin within two days of the event. The use of warfarin was contraindicated in future studies but other anticoagulants were allowed. Based on this study, the FDA granted accelerated approval to ibrutinib for the treatment of patients with MCL who had received at least one prior therapy.

Ibrutinib has also shown to be effective in relapsed/refractory CLL in a phase 2 trial in 85 heavily pre-treated patients with relapsed CLL who had received a median of four prior regimens [80]. This was also a high risk population with half of patients having either 17p13.1 or 11q22.3 deletions. The OR rate was 71% (95% CI, 60% to 80%) with a PFS of 75% at 26 months. The response was independent of clinical or genetic risk factors. Based on the results of this trial, the FDA granted accelerated approval to ibrutinib for treatment of patients with CLL who had received at least one prior therapy. A randomized phase 3 trial showed significant improvement in both PFS, OS, and response rate in patients with relapsed/refractory CLL who were treated with ibrutinib compared to ofatumumab [81].

Ibrutinib has also shown activity in activated B cell subtype DLBCL. DLBCL has two molecular subtypes, activated B cell subtype (ABC) and germinal center B-cell subtype (GCB), with ABC subtype showing worse outcomes with current therapy [82]. “Chronic active” B-cell receptor signaling has been found to be important for cell survival in ABC subtype of DLBCL [83,84]. A phase 2 trial investigated the activity of ibrutinib in relapsed/refractory DLBCL and tested the hypothesis that ibrutinib would be more active in ABC than GCB DLBCL [85]. The OR rate was 40% (10/25 patients, 95% CI, 21% to 61%) with an 8% CR rate (2/25 patients) in the ABC subtype. In contrast, the OR rate was 5.3% (1/19 patients) in the GCB subtype. This study supported the activity of ibrutinib especially in the ABC subtype of DLBCL although the sample size was too small to draw a conclusion.

A phase 1b study showed that ibrutinib was well tolerated when added to R-CHOP in untreated patients with B-cell NHL with an OR rate of 94% [86]. All 18 patients with DLBCL who received the recommended phase 2 dose had a response. For patients who were subtyped, five of seven patients (71%) with GCB subtype had a response and two of two patients (100%) with ABC subtype had a response. A randomized phase 3 trial of R-CHOP with and without ibrutinib in newly diagnosed patients with ABC (non-GCB) subtype DLBCL (NCT01855750) is currently in progress.

A phase 1/1b study evaluated the safety and efficacy of ibrutinib combined with rituximab and bendamustine (BR) in 48 patients with newly diagnosed MCL and relapsed/refractory indolent lymphoma, MCL, and DLBCL [87]. This study found an OR rate of 94% (76% CR) in MCL, 37% (31% CR) in DLBCL, and 90% (50% CR) in FL. Median PFS has not yet been reached. There were no dose limiting toxicities observed. The incidence of hematologic toxicities was similar to that seen in BR alone but there was an unexpected toxicity of grade 3 rash seen in 25% of patients in this study, which had not been observed in prior studies. The investigators concluded that this was due to ibrutinib but could be related to concurrent medications. The combination of BR and ibrutinib is promising in patients with MCL and FL but alternative combinations should be investigated in patients with DLBCL since it did not appear that BR added much to the efficacy of ibrutinib in this population.

Currently, a randomized phase 3 study comparing BR with and without ibrutinib in older patients with previously untreated MCL is in progress (NCT01776840). Another study is investigating R-CHOP or BR with or without ibrutinib in patients with relapsed/refractory indolent NHL (NCT01974440). There are also currently several trials looking at the combination of ibrutinib with rituximab and lenalodimide in NHL (NCT01955499, NCT 01829568).

A recent study showed that in addition to inhibiting BTK, ibrutinib also irreversibly binds IL-2 inducible T-cell kinase (ITK), which causes inhibition of Th2 activation [88]. This suggests that ibrutinib may have activity in other malignancies, such as T-cell NHL, in addition to B-cell malignancies. A phase 1 trial is currently evaluating the safety and efficacy of ibrutinib in relapsed/refractory T-cell NHL (NCT02309580).

There are also currently several other BTK inhibitors in early clinical trials.

### 4.2. Idelalisib (PI3Kδ Inhibitor)

Idelalisib is an oral specific and potent small molecule inhibitor of PI3Kδ. It was found to be generally well tolerated with promising efficacy and durable response in phase 1 trials in heavily pretreated patients with relapsed and refractory indolent NHL and CLL [70,71].

A randomized phase III placebo-controlled trial comparing rituximab with idelalisib to rituximab with placebo in 220 patients with relapsed CLL with coexisting medical conditions showed an improvement in OR, PFS, and OS in patients who received the combination without an increase in the rate of adverse events [89]. Based on these results, the FDA approved idelalisib for treatment of patients with relapsed CLL in combination with rituximab for those patients who are too medically frail to undergo standard chemotherapy.

A phase 2 trial evaluated the activity of idelalisib in 125 patients with indolent NHL who had either not had a response to rituximab and an alkylating agent or relapsed within six months of receiving those therapies [90]. This was a heavily pre-treated group of patients who had received a median of four prior therapies. The response rate was 57% (95% CI, 48% to 66%) with a 6% CR rate and a median duration of response and PFS of 12.5 months and 11 months, respectively. The most common grade 3 or higher adverse events were neutropenia (27%), transaminitis (13%), diarrhea (13%), and pneumonia (7%). Elevation in hepatic aminotransferases occurred in 47% of patients but grade 3 or higher elevations occurred in 13% and were reversible in all patients. 5% of patients discontinued treatment because of these lab abnormalities. Grade 3 or higher diarrhea or colitis occurred in 16% of patients at a median of six months after initiation of drug. These adverse events were often able to be managed with temporary interruption of drug treatment or dose adjustments. This study showed the overall favorable safety profile and promising and durable response of idelalisib in treatment of indolent NHL. Based on these results, the FDA granted accelerated approval to idelalisib in treatment of patients with relapsed FL and SLL who had received at least two prior systemic therapies.

Phase 3 trials investigating the efficacy and safety of idelalisib in combination with rituximab (NCT01732913) and rituximab/bendamustine (NCT01732926) in previously treated indolent NHL are currently in progress.

The activity of idelalisib in MCL was found to be more modest but still promising in this refractory disease. A phase 1 study of idelalisib in 40 patients with relapsed/refractory MCL showed an OR rate of 40% (95% CI, 24.9% to 56.7%) with a median duration of response of 2.7 months [91]. However, 22% of patients showed a 1-year PFS; therefore, the response was durable in some patients. The investigators postulated that the reason for brief remissions in MCL may be related to the disease’s ability to develop mechanisms of resistance, in this case, potentially by overexpression of the alpha subunit of PI3K.

A preclinical study evaluated the combination of ibrutinib and idelalisib in MCL and CLL, since both drugs target BCR signaling via different mechanisms, and found that together, the two agents showed more effective inhibition of BCR-controlled adhesion than either agent alone, supporting clinical study of these drugs in combination in B-cell malignancies [92].

### 4.3. Duvelisib (PI3Kγδ Inhibitor)

Duvelisib is a PI3K inhibitor which inhibits both the γ and δ isoform of PI3K. One mechanism of resistance to idelalisib could be related to the other PI3K family members that may become overexpressed to compensate for delta isoform inhibition [93]. Therefore, it has been theorized that dual inhibition of PI3K could help bypass this resistance.

A phase 1 study of 32 heavily pretreated patients with indolent B-NHL investigating the safety and efficacy of duvelisib showed an OR rate of 65% with a 25% CR rate [94]. The drug was fairly well tolerated but it did show a relatively high rate of grade 3 or higher elevation in liver enzymes (41% with median time to occurrence of 50 days), diarrhea (22% with a median time to occurrence of 124 days), and neutropenia (31%).

The safety and efficacy of duvelisib was also evaluated in 54 patients with relapsed/refractory CLL [95]. The best OR rate was 55% and the drug was generally well tolerated.

PI3γ and δ have also been shown to be expressed in cells with an important role in T-cell function. The safety and efficacy of duvelisib was investigated in 33 patients with relapsed or refractory T-cell NHL [96]. The OR rate was 42% (2 CR) with the most common grade 3 or higher adverse event again being transaminitis seen in 36% of patients. This supports the further investigation of this agent and other PI3K inhibitors in T-cell NHL.

There are several clinical trials investigating duvelisib including a phase 3 trial studying the combination of rituximab and duvelisib *vs*. rituximab and placebo in patients with previously treated FL (NCT02204982). 

### 4.4. TGR-1202 (PI3Kδ Inhibitor)

TGR-1202 is a selective PI3Kδ inhibitor which, due to differences in its structure, may have a lower rate of hepatotoxicity compared to other PI3Kδ inhibitors [97].

A phase 1 study evaluated the safety and efficacy of TGR-1202 with relapsed and/or refractory CLL and B cell lymphoma [98]. Eight out of nine evaluable patients with CLL have achieved a nodal PR. Five out of seven evaluable patients with FL have shown stable disease with two showing a PR. No hepatotoxicity or cases of colitis have been observed and the only grade 3 or higher adverse event to date is neutropenia (8%). The safety profile and response rates are promising and this study is still in progress.

Combination studies of TGR-1202 are also in progress with phase 1/1b safety and efficacy studies of TGR-1202 and ibrutinib in CLL or MCL (NCT02268851) and TGR1202 and BV in relapsed/refractory HL (NCT02164006).

### 4.5. Copanlisib (PI3Kαδ Inhibitor)

Copanlisib (Bay 80-6946) is a pan-Class I PI3K inhibitor which has activity against both PI3K-δ and PI3K-α isoforms. A phase 1 dose escalation study in patients with relapsed NHL established a maximum tolerated dose and showed an acceptable safety profile and promising activity [99]. Interim results of a phase II study of copanlisib in patients with relapsed/refractory indolent or aggressive lymphoma showed an overall response rate of 40% (20% CR) in FL, 67% (0% CR) in CLL, 83% (17% CR) in MCL, and 50% (0% CR) in peripheral T-cell lymphoma [100]. The drug was generally well tolerated and the most common grade 3 or 4 adverse events were hypertension (31%), neutropenia (16%), and hyperglycemia (13%). There is currently a randomized clinical trial investigating the combination of copanlisib with rituximab in indolent NHL (NCT02367040). There is also another randomized trial studying the activity of copanlisib in rituximab-refractory indolent NHL (NCT02369016).

### 4.6. Pacritinib (JAK2 Inhibitor)

Pacritinib (SB1518) is a small molecule inhibitor of JAK2 kinase and has shown preclinical activity in lymphoma. A phase 1 clinical trial investigating the safety and efficacy of pacritinib in 34 patients with relapsed or refractory HL or NHL showed an OR rate of 14% [72]. Treatment was well tolerated with mainly grade 1 or 2 toxicities. Although the OR rate was low, this study showed that potentially targeting the JAK/STAT pathway could be effective and raised questions about incorporating biomarker analysis that could predict response to therapy.

### 4.7. INCB040093 (PI3Kδ Inhibitor) and INCB039110 (JAK 1 Inhibitor)

The JAK-STAT and PI3K pathways play an important role in the survival of RS cells and are deregulated in classical HL [101]. The combination of agents targeting these pathways may have a synergistic effect. A phase 1 dose escalation trial of the combination of INCB040093, a PI3K*δ* inhibitor, and INCB039110, a selective JAK1 inhibitor, in patients with relapsed/refractory B-cell malignancies, including classical HL, showed an acceptable safety profile [102,103]. The ORR was 50% with one CR in the six patients with HL who received INCB040093 alone. In the nine patients with HL who received the combination of INCB040093 and INCB039110, the ORR was 67% with two CRs [103]. Given the promising results in patients with relapsed/refractory HL, a phase 2 trial studying INCB040093 alone and in combination with INCB039110 is in progress [104].

### 4.8. Navitoclax (Bcl-2 Inhibitor)

Navitoclax is a first generation BH3-mimetic which inhibits BCL2 and related proteins BCL-X_L_ and BCL-w, causing induction of apoptosis. A phase 1 study in 29 patients with relapsed and refractory CLL showed an OR rate of 35% and a median PFS of 25 months [74]. A parallel study in patients with relapsed and refractory lymphoid malignancies showed a durable PR in 10 of 46 patients with a median PFS of 455 days [105]. In particular, measurable tumor reduction was seen in six of 16 patients with FL. The major dose limiting toxicity for both studies was thrombocytopenia, which is caused by navitoclax’s activity against BCL-X_L_, which is required for platelet survival.

A phase 1 trial of navitoclax and rituximab postulated that the combination of these two agents with different mechanisms would increase efficacy without raising toxicity [106]. Twenty nine patients with relapsed or refractory CD20+ lymphoid malignancies including 12 patients with FL were enrolled in the study. Nine out of 12 patients with FL showed a response with five CR. All five patients with CLL/SLL achieved a PR. There was no apparent increase in adverse events compared to monotherapy of the drugs.

### 4.9. Venetoclax (Bcl-2 Inhibitor)

Although the clinical results for navitoclax seemed promising, the mechanism-related thrombocytopenia limited the ability to increase drug levels and potentially maximize efficacy. Navitoclax was re-engineered to create a more selective BCL-2 inhibitor, venetoclax (ABT-199), which also acts as a BH3 mimetic but shows higher specificity for BCL2 and lower specificity for BCL-X_L_, decreasing the rate of thrombocytopenia [107].

A phase 1 trial investigating safety and efficacy of venetoclax in 44 patients with relapsed/refractory NHL showed an OR rate of 48% including nine out of 12 patients with MCL showing a response [108]. Grade 3 or 4 thrombocytopenia occurred in 9% of patients but was not dose-dependent or dose-limiting, as opposed to that seen in navitoclax. Grade 3 laboratory tumor lysis syndrome was seen in one patient with bulky MCL and one patient with DLBCL. Given the promising activity of venetoclax in MCL, a combination phase 1 trial studying concurrent therapy of venetoclax and ibrutinib in relapsed/refractory MCL is currently ongoing (NCT02419560). There are also several studies evaluating the safety and efficacy of venetoclax in combination with cytotoxic chemotherapy in treatment of NHL including combination with BR (NCT0159422) and R-CHOP or CHOP plus obinutuzumab (NCT02055820).

A concurrent phase 1 trial of venetoclax in 93 patients with relapsed/refractory CLL and SLL with high risk features including 24% of patients with deletion of 17p, 59% with fludarabine refractory disease, and 32 of 42 patients with unmutated IGHV showed a response rate of 76% including 20% CR [109]. There was a 71% response rate in patients with deletion of 17p, 74% response rate in fludarabine refractory disease, and 74% response rate in patients with unmutated IGHV with a median duration of response of 20.5 months. Grade 3 or 4 thrombocytopenia was only seen in 7% of patients. 8% of patients had tumor lysis syndrome (including one grade 5) and modifications were made regarding TLS prophylaxis and monitoring once these toxicities were observed. Given the promising activity of venotoclax in high risk CLL, a phase 2 study investigating its activity in 17p deleted CLL including in the first line setting is currently ongoing (NCT01889186).

### 4.10. Alisertib (Aurora Kinase A Inhibitor)

Alisertib is a selective small molecule inhibitor of AAK which is being studied in both hematologic malignancies and solid tumors. A phase 1 study of 58 patients with relapsed or refractory lymphoid malignancies showed a PR in six patients (13%) and stable disease in 13 patients (28%) [75]. The most common tumor type in the study was NHL with 36 patients (16 patients with DLBCL, 10 patients with FL). This was a heavily pretreated group with 76% of patients receiving three or more prior lines of therapy. The most frequent grade 3 or higher drug-related toxicities were hematologic with 45% of patients having neutropenia and 28% of patients having thrombocytopenia.

A phase 2 trial of 48 patients with relapsed/refractory aggressive B and T-cell NHL showed an OR rate of 27% [110]. Three out of 21 patients with DLBCL, three out of 13 patients with MCL, one of one with Burkitt’s lymphoma, two of five with transformed FL, and four of eight with non-cutaneous T-cell lymphoma showed a response. Of interest, there was no association between clinical response and AAK expression in tumor samples which encourages the search for other potentially predictive biomarkers.

Given the promising response in peripheral T cell lymphomas, a phase 2 trial further investigating the efficacy of alisertib in patients with relapsed or refractory peripheral T-cell lymphoma was conducted, with an OR rate of 30% (95% CI, 9% to 61%) in peripheral T cell lymphoma not-otherwise-specified subtypes [111]. The investigators evaluated several biomarkers including AAK expression and did not find any association with response. Given the promising results of this study, there is currently a randomized phase III trial comparing alisertib to investigator’s choice in relapsed or refractory peripheral T cell lymphoma (NCT01482962).

In the phase 2 study across all lymphomas described above, there was also some activity in aggressive B-cell lymphomas [110]. One patient with Burkitt’s lymphoma and one patient with FL with double hit features (co-overexpressing Myc and BCL-2) responded to treatment. Aurora kinases are upregulated by MYC and seem to be important in MYC-driven B-cell lymphoma. A pre-clinical trial in mice showed that alisertib worked synergistically with rituximab and vincristine to demonstrate a strong and durable response in double hit DLBCL [112]. A phase 1/2 study of alisertib in patients with relapsed or refractory DLBCL/transformed FL in combination with rituximab and vincristine is ongoing (NCT01397825).

## 5. Conclusions

There have been a large number of novel agents approved and currently being investigated for treatment of HL and NHL, including many targets and agents not included in this review. Many of these agents have shown durable responses with improved safety profiles. This has shifted the treatment paradigm to non-cytotoxic therapies with the development of novel antibodies and antibody-drug conjugates, immunotherapy, and small molecule inhibitors.

The standard of care for lymphoma will continue to change as new targeted agents gain FDA approval. Future challenges include determining the most optimal approach to incorporate these new therapies in our current treatment algorithms (first line, in combination with cytotoxic chemotherapy, *etc.*), managing the high cost of novel agents, and developing biomarkers that can predict which patients would best respond to these novel therapies as we enter an era of more personalized medicine.
